# Systematic Profiling of Alternative mRNA Splicing Signature for Predicting Glioblastoma Prognosis

**DOI:** 10.3389/fonc.2019.00928

**Published:** 2019-09-24

**Authors:** Xueran Chen, Chenggang Zhao, Bing Guo, Zhiyang Zhao, Hongzhi Wang, Zhiyou Fang

**Affiliations:** ^1^Anhui Province Key Laboratory of Medical Physics and Technology, Center of Medical Physics and Technology, Hefei Institutes of Physical Science, Chinese Academy of Sciences, Hefei, China; ^2^Department of Molecular Pathology, Hefei Cancer Hospital, Chinese Academy of Sciences, Hefei, China; ^3^University of Science and Technology of China, Hefei, China

**Keywords:** alternative splicing (AS) events, glioblastoma (GBM), overall survival, disease-free survival, prognostic predictor

## Abstract

Emerging evidence suggests that alternative splicing (AS) is modified in cancer and is associated with cancer progression. Systematic analysis of AS signature in glioblastoma (GBM) is lacking and is greatly needed. We profiled genome-wide AS events in 498 GBM patients in TCGA using RNA-seq data, and splicing network and prognostic predictor were built by integrated bioinformatics analysis. Among 45,610 AS events in 10,434 genes, we detected 1,829 AS events in 1,311 genes, and 1,667 AS events in 1,146 genes that were significantly associated with overall survival and disease-free survival of GBM patients, respectively. Five potential feature genes, S100A4, ECE2, CAST, ASPH, and LY6K, were discovered after network mining as well as correlation analysis between AS and gene expression, most of which were related to carcinogenesis and development. Multivariate survival model analysis indicated that these five feature genes could classify the prognosis at AS event and gene expression level. This report opens up a new avenue for exploration of the pathogenesis of GBM through AS, thus more precisely guiding clinical treatment and prognosis judgment.

## Highlights

- Alternative splicing (AS) events may act as prognosis predicting factors for GBM
- S100A4, ECE2, CAST, ASPH, and LY6K were correlated between AS and gene expression
- A survival model with five feature genes efficiently classified GBM prognosis

## Introduction

Tumors derived from the neural epithelium, generally called gliomas, account for 40–50% of brain tumors, and are the most commonly observed intracranial malignant tumors (1, 2). Glioblastoma (GBM) is a rapidly growing glioma that develops from the healthy neuroglial cells that support the nerve cells in the brain (including astrocytes and oligodendrocytes) (3, 4). As a devastating brain cancer, GBM is the most invasive glioma, which can rapidly grow and generally diffuse to adjacent brain tissues. Inability to detect GBM at an early stage and its post-operative recurrence are largely responsible for these low survival rates (5–7). Currently, there are no reliable methods for detecting early-stage GBM and evaluating prognosis for GBM patients.

Cancer is, in many ways, a genetic disease and recent studies have focused on differences in molecular profiling of gene expression patterns to uncover diagnostic and prognostic markers as well as novel therapeutic targets (8–10). However, these studies, although with promising results, focus on alterations mainly at the gene expression level while transcript architecture regulated by alternative splicing (AS) is ignored.

High-throughput sequencing studies showed that more than 90% of human genes are subjected to AS (11, 12). AS of pre-mRNA is a universal mechanism to generate mRNA isomers using a limited set of genes. AS is a process in which the introns of a majority of human multi-exon genes are deleted, and specific exons are alternatively included or excluded (13, 14). Apart from protein diversity, the mRNA isomer translation level can also be down-regulated by introducing AS, leading to the degradation of the early termination codon (15). Aberrant AS is implicated in a variety of diseases, such as neurodegenerative diseases and cancers (16, 17). Cancer-specific alterations in splice site selection affect genes controlling cellular proliferation (e.g., CD44, Cyclin D1, HER2, and H-Ras), invasion (e.g., CD44, Ron, and MENA), angiogenesis (e.g., VEGF), apoptosis (e.g., Fas, Bcl-x, and caspase-2), and multi-drug resistance (e.g., MRP-1 and p53) (18–20). Therefore, AS provides a critical and flexible layer of regulation on many biological processes, and profiling of AS signature may provide potential biomarkers for cancer.

Since 1997, increasing number of studies have suggested associations between AS events and patient survival in GBM (21–23). However, systematic survival analyses of AS in GBM have not been reported yet and are urgently required. Here, we use The Cancer Genome Atlas (TCGA) RNA sequencing data to gather key genes that affect the prognosis of GBM based on genome AS event analysis and to classify GBM samples into high and low risks using the prognosis model constructed according to gene expression profiles and AS events.

## Materials and Methods

### Alternative Splicing Event Curation From TCGA RNA-seq Data

RNA sequencing data of TCGA GBM cohort were downloaded from TCGA data portal (https://tcga-data.nci.nih.gov/tcga/). The RNA-Seq expression profile Fragments Per Kilobase Million (FPKM) dataset was downloaded and further converted into Transcripts Per Million (TPM) data; at the same time, the ID was transformed using the genome file of GENCODE (GRCh38.p2), and the protein encoding genes were obtained (24). To generate the AS profiles for each patient, SpliceSeq, a *java* application that unambiguously quantifies the inclusion level of each exon and splice junction (25), was used to evaluate the mRNA splicing patterns for patients in the GBM cohort. A total of 153 common samples in both TCGA SpliceSeq and RNA-Seq were enrolled in this study, and a total of 19,754 genes with expression values were obtained as the total gene set in this study. The Percent Spliced In (PSI) value, rating from zero to one and commonly used to quantify AS events (25), was calculated for seven types of AS events: Exon Skip (ES), Mutually Exclusive Exons (ME), Retained Intron (RI), Alternate Promoter (AP), Alternate Terminator (AT), Alternate Donor site (AD), and Alternate Acceptor site (AA).

### Survival Analysis

A total of 597 GBM patients with at least 30 days of overall survival (OS) were included in this study. Patients were then divided into two groups by median cut for each parameter, respectively. Univariate Cox regression followed by multivariate Cox regression was performed to determine independent prognostic factors and to build prediction models. The efficiencies of each prediction model were compared using survival ROC package (version 1.0.3) in *R* (version 3.3.0), which allows for time dependent receiver-operator characteristic (ROC) curve estimation with censored data. The area under the curve (AUC) of ROC curve was calculated for each model at 2,000 days of OS, since fewer events occurred after 2,000 days (see Kaplan-Meier curves). All reported *p*-values were two-sided.

### UpSet Plot and Gene Network Construction

UpSet plot, a novel visualization technique for quantitative analysis of interactive sets, was used to analyze the intersections between the seven types of alternative splicing (26). To observe the gene associations among the various types of AS events that were markedly correlated with prognosis, their corresponding genes were mapped to the String database, respectively. Then, the interactions of these genes were obtained using the score of >0.4, and Cytoscape (version 3.4.0) was used for visualization (27). Kyoto Encyclopedia of Genes and Genomes (KEGG) pathway enrichment analyses were conducted for the identified differentially spliced genes via DAVID (28). *p* < 0.05 indicates statistical significance.

### Colony Formation Assay

After receiving informed consent, GBM specimens were obtained from patients undergoing surgery at the Hefei Cancer Hospital, Chinese Academy of Sciences in accordance with the Institutional Review Boards. Within hours after surgical removal, tumor specimens were enzymatically dissociated into single cells, following previously reported procedures (29). The cells were plated at a seeding density of 500 cells/plate in a 10-cm plate with or without 6 Gy radiotherapy + 200 μM temozolomide, grown for 10 days in a standard growth medium, and washed with PBS. The cells were fixed in cold methanol for 20 min, washed, and stored. Fixed cell colonies were visualized by incubating the cells with 0.5% (w/v) crystal violet for 0.5 h. Excess crystal violet was removed by washing with PBS. The visible colonies, consisting of ≥50 cells, were counted. Differences in means were considered statistically significant when *p* < 0.05 using a two-tailed *t*-test.

## Results

### Alternative Splicing Profiles in TCGA GBM Cohort

**Figure 1:**
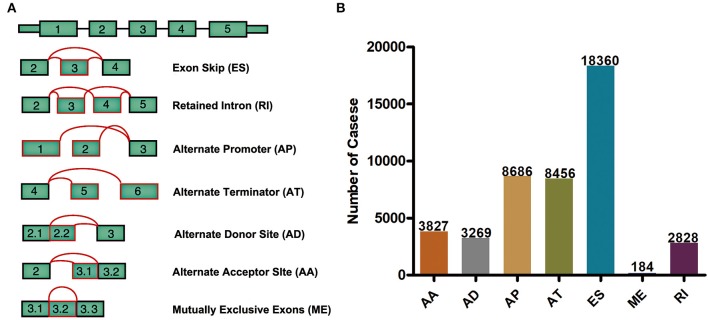
Overview of seven types of AS in this study. **(A)** Illustrations for 7 different AS events. **(B)** Number of AS events from the 498 GBM patients.

Integrated mRNA splicing event profiles were analyzed in depth for 498 GBM patients from TCGA. Seven types of AS events, including Exon Skip (ES), Mutually Exclusive Exons (ME), Retained Intron (RI), Alternate Promoter (AP), Alternate Terminator (AT), Alternate Donor site (AD), and Alternate Acceptor site (AA), are illustrated in Figure 1A. A total of 45,610 AS events form 10,434 genes were detected, indicating that one gene might have almost four AS events on average. Typically, ES was the major type, and ES events accounted for almost 1/2 of all alternative splicing events (Figure 1B).

### Prognosis-Associated Alternative Splicing Events in the TCGA GBM Cohort

**Figure 2:**
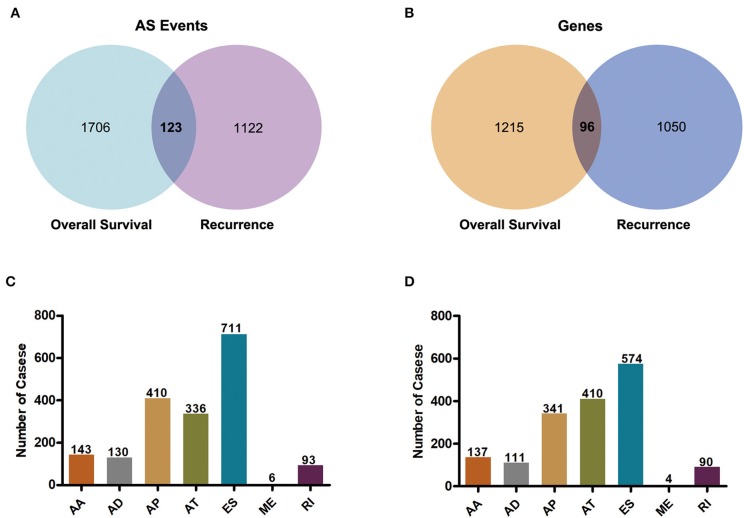
Overview of prognosis-related alternative splicing events in GBM. **(A)** Venn diagram of AS events of genes significantly related to overall survival and those related to recurrence after radio- and chemo-therapy. **(B)** Venn diagram of gene intersections in AS events of genes significantly related to overall survival and those related to recurrence after radio- and chemo-therapy. **(C)** Histogram of the 7 types of AS events that were markedly correlated with overall survival in the gene AS events. **(D)** Histogram of the 7 types of AS events that were remarkably correlated with recurrence after radio- and chemo-therapy for gene AS events.

To observe the relationships between AS events and prognosis of GBM patients, all clinical follow-up data of diseases were integrated into Supplementary Table 1, and univariate survival analysis was performed for 45,610 AS events to examine the relationships between these AS events and the prognosis of GBM patients. When selecting *p* < 0.05, a total of 1,829 alternative splicing (AS) events involving 1,311 genes that were remarkably correlated with overall survival (OS) were obtained. Additionally, 1,667 AS events, covering 1,146 genes that were markedly correlated with disease-free survival (DFS) were acquired, as displayed in Supplementary Table 2. Besides, there were 123 intersections between AS events that were significantly correlated with OS and DFS, as presented in Figure 2A. Among them, there were 96 intersected genes among all the involved genes, as shown in Figure 2B, suggesting that there was consistency between genes involved in OS and DFS, to a certain extent. Most of these 96 genes were significantly associated with malignant progression and prognosis of GBM patients, including DKK3, NOTCH2NL, and HDAC9. We counted the AS events that were markedly correlated with OS and found that only the frequency of AP events (4.72%; 410/8,686) exceeded 4.01% (1,829/45,610), and that of the remaining events was under 4.01% (Figure 2C). Furthermore, among the AS events related to DFS, the frequency of AT events (4.85%; 410/8,456) and AP events (3.95; 341/8,686) exceeded the average level of 3.65% (1,667/45,610) (Figure 2D). Thus, these results suggested that there might be more AP and AT events associated with prognosis compared to ES events.

We noticed that one gene might have two or more events that were significantly associated with patient survival. Thus, Upset plot, a more scalable alternative to Venn diagram for visualizing intersecting sets, was generated and shown in Supplementary Figure 1. Interestingly, one gene might have up to three types of AS events that were significantly associated with patient survival. For example, AP, AD, and AA events in the DLGAP1 gene were significantly associated with OS in the GBM cohort, while ES, RI, and AA events in the *NBPF11* gene were significantly associated with DFS in the GBM cohort.

### Gene Interaction Network in Seven Types of Prognosis-Associated Alternative Splicing Events

To observe gene associations among different types of AS events that were apparently correlated with survival, the genes were mapped to the String database, and gene interactions were obtained using a score of >0.4 and visualized by Cytoscope. Cytoscape analysis of gene network revealed important cancer pathways including hub genes at NFGR, MAPK3, and SMAD7 for OS, and hub genes at FOXP1, MAP2K5, FGFR1, and NOTCH2 for DFS (Supplementary Figure 2).

To observe gene function in various types of AS events that were significantly correlated with survival, the AS genes in each AS event type that were significantly correlated with survival were also analyzed by KEGG enrichment. The results are shown in Supplementary Figure 3, from which it can be seen that these genes were enriched in multiple disease-related pathways, suggesting that these genes are involved in numerous biological functions. For example, KEGG enrichment of genes in RI events significantly correlated with OS, showing that these genes are involved in base excision repair. Moreover, KEGG enrichment of genes in AS events significantly correlated with DFS, indicating that the genes in ME events are involved in basal transcription factors, viral carcinogenesis, and nucleotide excision repair and that genes in AA events are involved in citrate cycle. These results indicated that the genes in AS events were mainly enriched in DNA damage repair because the mechanism of radio- and chemo-therapy for GBM patients involves damage to tumor DNA, leading to apoptosis.

### Analysis of the Prognosis Factors of GBM Alternative Splicing Events

**Figure 3:**
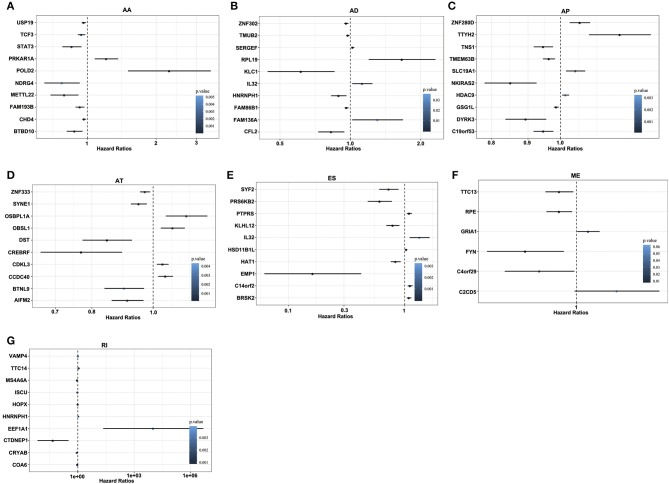
Forest plots of survival-associated AS events in GBM. **(A–G)** Hazard ratios of top 10 survival-associated AA, AD, AP, AT, ES, ME, and RI events.

For each AS event, GBM patients were divided into two groups based on the PSI value (median cut). In the univariate Cox regression, a total of 1,829 and 1,667 AS events were significantly associated with OS and DFS of GBM patients (*p* < 0.05), respectively. For each type of AS events, the Hazards Ratios (HRs) of the top 10 most significant AS events (if available) were selected and visualized in Figure 3 and Supplementary Figure 4. Interestingly, most of these survival associated AS events were favorable prognostic factors (HR <1). For example, there were eight genes in OS-associated AA splicing pattern with a hazard ratio (HR) of <1, and 2 with HR of > 1; consistently, there were seven genes in DFS-related splicing with HR of <1 and 3 with HR of >1.

**Figure 4:**
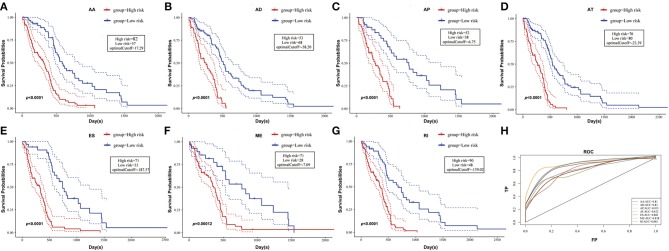
Kaplan-Meier plots and ROC curves of prognostic predictor for GBM patients. **(A–G)** Kaplan-Meier curves for prognostic prediction model built with one type of survival-associated AS event for GBM patients, respectively. The red line indicates a high-risk group, while the blue line indicates a low risk group. **(H)** ROC curves with AUC of prognostic predictor built by one type of all seven types of survival-associated AS events in GBM.

To observe whether the selective AS events could be used as prognosis factors, the 10 most significant genes of each AS pattern were selected from all prognosis-related AS events for multivariate regression model analysis. As could be seen from Figure 4, the seven types of AS events had large areas under the curve (AUC) for prognosis classification, among which the AP and ES patterns of AS displayed the best overall survival, while ES and RI patterns had the best performance among AS significantly correlated with recurrence after radio- and chemo-therapy, as shown in Supplementary Figure 5, revealing that AS might serve as a new prognosis classification method.

### Relationships Between Gene Expression Profile and Prognosis in Prognosis-Associated AS Events

To investigate the relationships between gene expression and prognosis in AS events that were notably correlated with prognosis, the TCGA RNA-Seq expression profile data were employed for univariate survival analysis of each gene. Finally, it was discovered that among the 1,311 overall survival-related AS genes, the expression of 113 genes was related to OS; whereas among the 1,146 AS genes related to DFS, the expression of 87 genes was significantly correlated with DFS. Furthermore, correlation analysis was performed on these 113 OS genes with the corresponding AS events using Pearson correlation coefficient. Finally, 55 genes (48.67%) significantly correlated with AS were obtained (*p* < 0.05), indicating that the AS events of ~50% of the genes were significantly associated with their expression. In addition, of the 87 genes related to DFS, 63 (72.41%) were markedly correlated with AS, demonstrating that the AS events of most genes were markedly associated with their expression.

**Figure 5:**
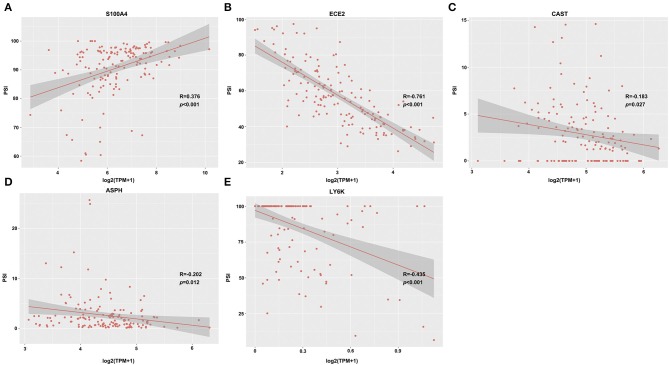
Representative dot plots of correlations between expression of 5 feature genes [S100A4 **(A)**, ECE2 **(B)**, CAST **(C)**, ASPH **(D)**, and LY6K **(E)**] and PSI values of AS events (*p* < 0.05).

Genes with Pearson correlation coefficient between gene expression profiles and AS events of > 0.2 or < −0.2 were selected, including 35 OS-related and 25 DFS-related genes. Of the 35 OS-related genes, 5 were related to DFS (including S100A4, ECE2, CAST, ASPH, and LY6K), and their correlations with the transcriptome levels are presented in Figure 5. It can be seen that S100A4 showed a positive correlation, while the remaining displayed a negative correlation. The role of S100A4 in controlling cell proliferation, cancer invasion, and metastasis has been extensively studied in numerous laboratories (PMID: 9703888) (30, 31). ASPH has been reported as a potential therapeutic target for malignant glioma (PMID: 27981247) (32, 33), and LY6K is a novel bladder cancer molecular target that integrated genome-wide analysis (PMID: 21063397) (34, 35). Notably, AP event in gene S100A4, AT event in genes ECE2 and ASPH, ES event in gene CAST, and RI event in gene LY6k were significantly associated with OS (or DFS) of GBM patients (Supplementary Table 3), suggesting that the potential mechanisms of AS events have an impact on survival.

### Construction of the Prognosis Model

**Figure 6:**
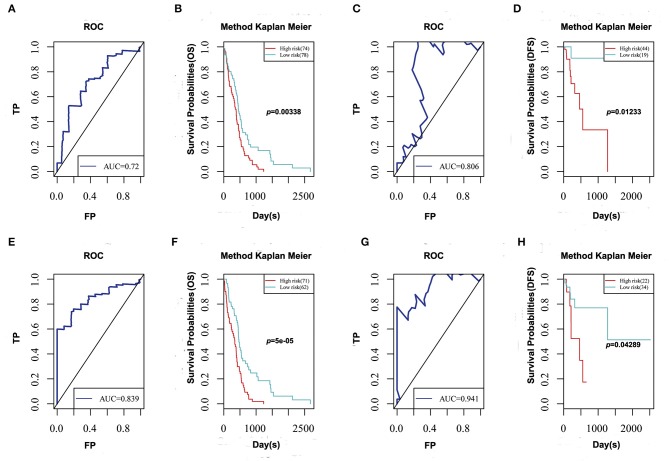
ROC curve and Kaplan-Meier plot of prognostic predictor for GBM patients. **(A,C)** ROC curve with AUC of prognostic predictor related to overall survival **(A)** and disease-free survival **(C)** built by alternative events of 5 feature genes in GBM. **(B,D)** Kaplan-Meier curves of prognostic predictor related to overall survival **(B)** and disease-free survival **(D)** built by alternative events of 5 feature genes in GBM. **(E,G)** ROC curve with AUC of prognostic predictor related to overall survival **(E)** and disease-free survival **(G)** built by alternative events and transcriptome levels of 5 feature genes in GBM. **(F,H)** Kaplan-Meier curves of prognostic predictor related to overall survival **(F)** and disease-free survival **(H)** built by alternative events and transcriptome levels of 5 feature genes in GBM.

To determine prognosis-predicting indices that were suitable for GBM patients and to facilitate clinical practice, five feature genes were selected to construct a multivariate survival model to observe the classification of prognosis by these five feature genes at AS event and expression profile levels (Figure 6). The prognostic predictor with these five feature genes on AS events indeed showed favorable performance in distinguishing good or poor survival in GBM patients, with great AUC. Notably, the final prognostic predictor with these five feature genes at AS event and expression profile levels presented better prognosis classification effects. ROC curves confirmed that the final prognostic predictor with these five feature genes at both AS event and expression profile levels had better efficiency than the model built on only the alternative splicing events.

Indeed, these two risk subgroups might reflect different GBM intrinsic tumor subtypes. In particular, the high-risk subgroup was highly enriched for classical and mesenchymal GBM. On the other hand, more neural and proneural GBM were found in the low risk subgroup (Supplementary Table 4). Moreover, these two subgroups were significantly related to some molecular genetic features, particularly in TP53 and IDH1 mutant statuses, and 1p/19q co-deletion (Supplementary Table 4). The high-risk subgroup (62%) exhibited more p53 mutations than did the low-risk subgroup (9.7%). Moreover, the high-risk subgroup (88%) contained the largest proportion of wild-type IDH1. 1p/19q co-deletion was higher in the high-risk subgroup (71%) than in the low-risk subgroup (43%). Thus, the two risk subgroups based on AS event and expression profile may reflect changes in some molecular genetic features, and these results also indicated that these five genes might serve as prognostic markers of GBM.

Based on the final prognostic predictor, 12 clinical GBM specimens were divided into low-risk (*n* = 5) and high-risk (*n* = 7) groups (Supplementary Figures 6A,B). The results of colony formation assays showed that the inhibitory effect of radiotherapy (6 Gy) and temozolomide (200 μM) on high-risk GBM specimen-derived cells was weaker than that on low-risk GBM specimen-derived cells (Supplementary Figures 6A–C). Consistent with these, there was an inverse correlation between the risk level and OS (*p* < 0.0499), with AUC of 0.8750 (Supplementary Figures 6D,E). Consistent with these, the risk level displayed a positive correlation with migration ability (Supplementary Figure 6F), neurosphere formation ability (Supplementary Figure 6G), and the expression levels of stemness markers (Supplementary Figures 6H,I) of GBM cells derived from these 12 GBM patients. Taken together, this prognostic predictor showed great promise in application in clinical practice.

## Discussion

Aberrant pre-mRNA AS has been widely established as a novel contributor to cancer development (36, 37). Although a number of cancer-specific mRNA isoforms have been identified, there is a lack of understanding of AS event profiles and their functional pathways. With the rapid development of high-throughput sequencing and bioinformatics methods, a more comprehensive overview of AS in GBM can be obtained. Published genome-wide studies on AS in GBM mainly focus on identifying “cancer-specific” AS events by comparing cancer tissues with normal controls. Here, we mainly performed a systematic identification and analysis of prognosis-associated AS events in 498 GBM patients in TCGA. Moreover, combined survival and correlation network analysis between AS events and their expression profiles offered an approach to address the underlying mechanism of AS events involved in patient prognosis. Finally, we showed that prognosis-associated AS events and their expression could be used to construct prognostic predictors with high performance for risk stratification in GBM; these predictors showed promise for application in clinical practice.

Diverse splicing patterns in one gene lead to a variety of isoforms, which makes AS and its regulation mechanism more complex in cancer (38–40). In this study, AS signatures in 498 GBM patients were profiled followed by integrated survival analyses with powerful prognosis predictors being built. A total of 45,610 AS events of 10,434 genes were detected. In addition, GBM generates the largest number of ESs and smallest number of MEs. The 10 most significant genes among the AS types were selected for multivariate regression model analysis to observe their ability to classify prognosis. The prediction model built with ES events showed the highest efficiency in distinguishing good or poor outcome of GBM patients among all the seven types.

The AS genes that evidently correlated with prognosis in each type were analyzed by KEGG enrichment analysis, revealing important cancer pathways, such as RI of base excision repair for patient survival; ME of viral carcinogenesis, basal transcription factors, nucleotide excision repair, and Fc gamma R-mediated phagocytosis; and AA of TCA cycle for tumor recurrence. Further investigations into how alternative splicing modulates these procedures are required in the future.

The TCGA RNA-Seq expression profile data were used for univariate survival analysis of each gene to observe the relationships between gene expression and prognosis in AS events that were markedly correlated with prognosis. Furthermore, the influence of expression profiles of genes subjected to AS events on prognosis was also examined. Five potential feature genes, including S100A4, ECE2, CAST, ASPH, and LY6K, were discovered after network mining as well as correlation analysis between alternative splicing and gene expression. A majority of these genes were related to cancer onset and development. For example, S100A4, an important member of S100 family proteins, functions to increase tumor progression and metastasis through TGFβ/Smad, NFkB, and Wnt/β-catenin signaling pathways (41, 42). Aspartate-β-hydroxylase (ASPH) is expressed at high levels in several malignant neoplasms of distinct histogenesis, and at very low levels or not at all in most normal cells and tissues. GBM was associated with the highest levels of ASPH, more abundantly distributed in hypoxic than in normoxic tumor regions (33). Multivariate survival model analysis indicated that these five feature genes could efficiently classify prognosis at AS event and gene expression levels. Indeed, the results of 12 clinical GBM specimens also confirmed that this prognostic predictor has great promise in clinical applications. The GBM cells derived from GBM patients at the high risk level exhibited more malignant behavior than did those derived at the low risk level. However, there was a lack of *in vivo* models of GBM involving the five AS-related genes to further demonstrate the application prospects of the final prognosis prediction. The further study will focus on the change of AS event and expression level of these five AS-related genes before or after post-radiation and TMZ combination treatment.

Given the high prevalence of splicing defects in cancer, small molecule modulators of RNA processing represent a potentially promising novel therapeutic strategy in cancer treatment. A recent review summarized that there were a number of small molecule modulators, including the earliest FR901464, showing promising effects in cancer therapy (43, 44). Knockdown of S100A4 may be a valuable therapeutic target because there is a positive correlation between AS events and its expression level. Although AS events of the genes ECE2, CAST, ASPH, and LY6K were negatively correlated with their expression, they would also offer more therapeutic strategies. For example, alternative terminator (AT) is the main type of AS event in the gene, ASPH, in GBM and might result in the upregulation of Humbug's transcriptome levels. Both dysfunction of splicing factor and knockdown of the truncated isoform of ASPH, Humbug, may be potential therapeutic strategies. Thus, our study also provided a number of potential targets for GBM therapy.

In summary, we reported that prognosis-associated AS events were ideal for prognostic predictor construction, and our final model performed well in risk stratification for GBM patients. A series of cancer-specific and prognosis-associated AS events were identified to provide potential therapeutic targets for GBM. Interaction network and functional connections were also constructed, which would enrich our understanding of the role of RNA alternative splicing in the tumorigenesis of GBM.

## Data Availability Statement

The raw data supporting the conclusions of this manuscript will be made available by the authors, without undue reservation, to any qualified researcher.

## Ethics Statement

This study was reviewed and approved by the Institutional Review Board of the Animal Use and Care Committees at Hefei Institutes of Physical Science, CAS.

## Author Contributions

XC and ZF conceived and designed the experiments. CZ, BG, and ZZ collected the data. XC and CZ performed the analysis, prepared, and edited the manuscript. XC, BG, HW, and ZF participated in the discussion of the algorithm. All authors have read, approved the final manuscript and have agreed to publish this manuscript.

### Conflict of Interest Statement

The authors declare that the research was conducted in the absence of any commercial or financial relationships that could be construed as a potential conflict of interest.

## Supplementary Material

Supplementary Table 1 Clinical follow-up data of diseases prognosis.

Supplementary Table 2 Genes associated with prognosis-associated AS events.

Supplementary Table 3 Risk score of OS (or DFS) of GBM patients based on gene expression and PSI values of AS events of 5 feature genes.

Supplementary Table 4 Relationship between the clinical characteristics and risk level of GBM patients.

Supplementary Figure 1 Upset plot of interactions between the seven types of prognosis-associated AS events in GBM. **(A)** Gene distribution of the seven different AS events that were significantly correlated with overall survival; **(B)** gene distribution of the seven different AS events that were significantly correlated with recurrence after radio- and chemo-therapy.

Supplementary Figure 2 Gene network of prognosis-associated AS in GBM generated by Cytoscape. **(A)** Gene interaction network in the seven different AS events that were significantly correlated with overall survival. **(B)** Gene interaction network in the seven different AS events that were significantly correlated with recurrence after radio- and chemo-therapy.

Supplementary Figure 3 KEGG analysis results of genes in the seven AS events associated with patient prognosis. **(A)** KEGG enrichment of genes in the seven different AS events that were significantly correlated with overall survival. **(B)** KEGG enrichment of genes in the seven different AS events that were significantly correlated with recurrence after radio- and chemo-therapy.

Supplementary Figure 4 Forrest plots of recurrence-associated AS events in GBM. **(A–G)** Hazard ratios of top 10 recurrence associated AA, AD, AP, AT, ES, ME, and RI events.

Supplementary Figure 5 Kaplan-Meier plots and ROC curves of prognostic predictor for GBM patients. **(A-G)** Kaplan-Meier curves of prognostic predictor built with one type of recurrence associated AS events for GBM patients, respectively. Red line indicates high-risk group, while blue line indicates low-risk group. **(H)** ROC curves with AUC of prognostic predictor built by one type of all seven types of recurrence-associated AS events in GBM.

Supplementary Figure 6 Application in clinical practice of the final prognostic predictor on five feature genes. **(A-C)** Colony formation assay for 12 GBM specimen-derived cells under radiotherapy (6 Gy) and temozolomide treatment (200 μM). **(D)** ROC curve with AUC under the final prognostic predictor. **(E)** The Kaplan-Meier curve of 12 GBM patients. Red line indicates high-risk group, while blue line indicates low-risk group, based on the final prognostic predictor. **(F)** The proportion of migrated GBM cells derived from GBM patients. **(G)** The rate of neurosphere formation of GBM cells derived from GBM patients. **(H,I)**, SOX2 **(H)** or CD133 **(I)** mRNA level of GBM cells derived from GBM patients determined by RT-PCR. Data are presented as means ± SEM (^*^*p* < 0.05; ^**^*p* < 0.01; ^***^ *p* < 0.001).

## Abbreviations

AA: alternate acceptor site
AD: alternate donor site
AP: alternate promoter
AS: alternative splicing
AUC: area under curve
AT: alternate terminator
ES: exon skip
GBM: glioblastoma
HR: hazard ratio
ME: mutually exclusive exons
mRNA: messenger RNA
OS: overall survival
PSI: Percent Spliced In
RI: retained intron
TCGA: The Cancer Genome Atlas.
