# Supplementary material for: Systematic Profiling of Alternative mRNA Splicing Signature for Predicting Glioblastoma Prognosis
Source: Front Oncol. 2019 Sep 24;9:928. doi: 10.3389/fonc.2019.00928 (PMC6769083; doi:10.3389/fonc.2019.00928)
Supplement: Supplementary Figure 1 — Upset plot of interactions between the seven types of prognosis-associated AS events in GBM. (A) Gene distribution of the seven different AS events that were significantly correlated with overall survival; (B) gene distribution of the seven different AS events that were significantly correlated with recurrence after radio- and chemo-therapy. [file Data_Sheet_1.PDF]

Supplementary Figure 1

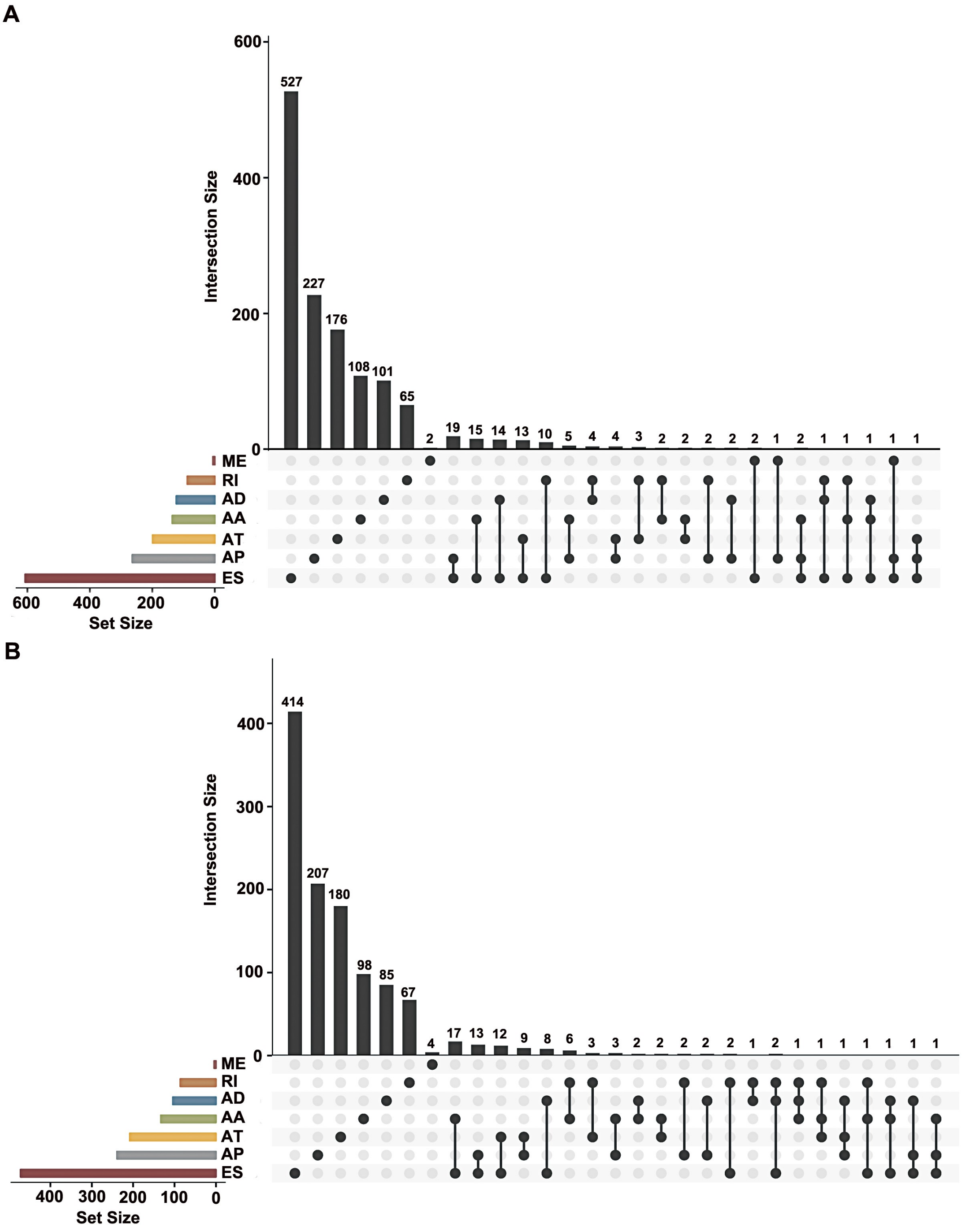

Supplementary Figure 1. Upset plot of interactions between the seven types of prognosis-associated AS events in GBM. A. Gene distribution of the 7 different AS events that were significantly correlated with overall survival. B. Gene distribution of the 7 different AS events that were significantly correlated with recurrence after radio- and chemo-therapy.

Supplementary Figure 2

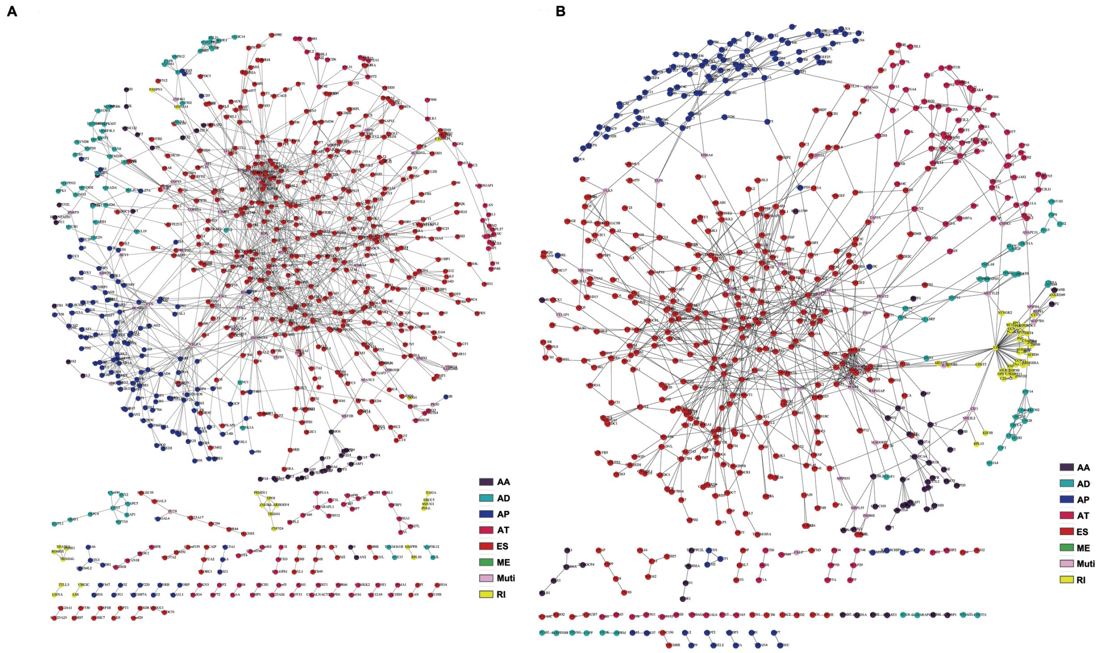

Supplementary Figure 2. Gene network of prognosis-associated AS in GBM generated by Cytoscape. A, Gene interaction network in the 7 different AS events that were significantly correlated with overall survival. B, Gene interaction network in the 7 different AS events that were significantly correlated with recurrence after radio- and chemo-therapy.

Supplementary Figure 3

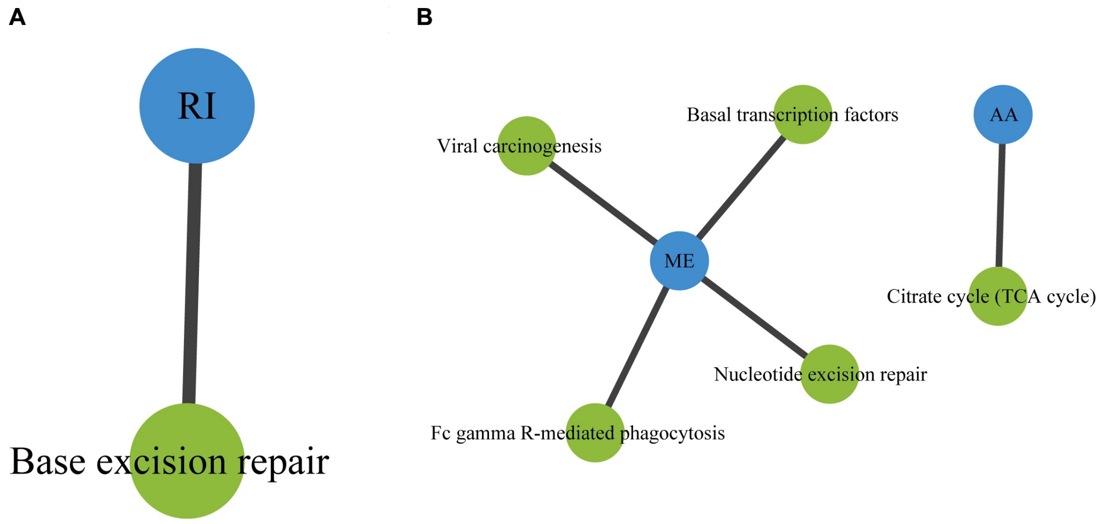

Supplementary Figure 3. KEGG analysis results of genes in the seven AS events associated with patient prognosis. A. KEGG enrichment of genes in the 7 different AS events that were significantly correlated with overall survival. B. KEGG enrichment of genes in the 7 different AS events that were significantly correlated with recurrence after radio- and chemo-therapy.

Supplementary Figure 4

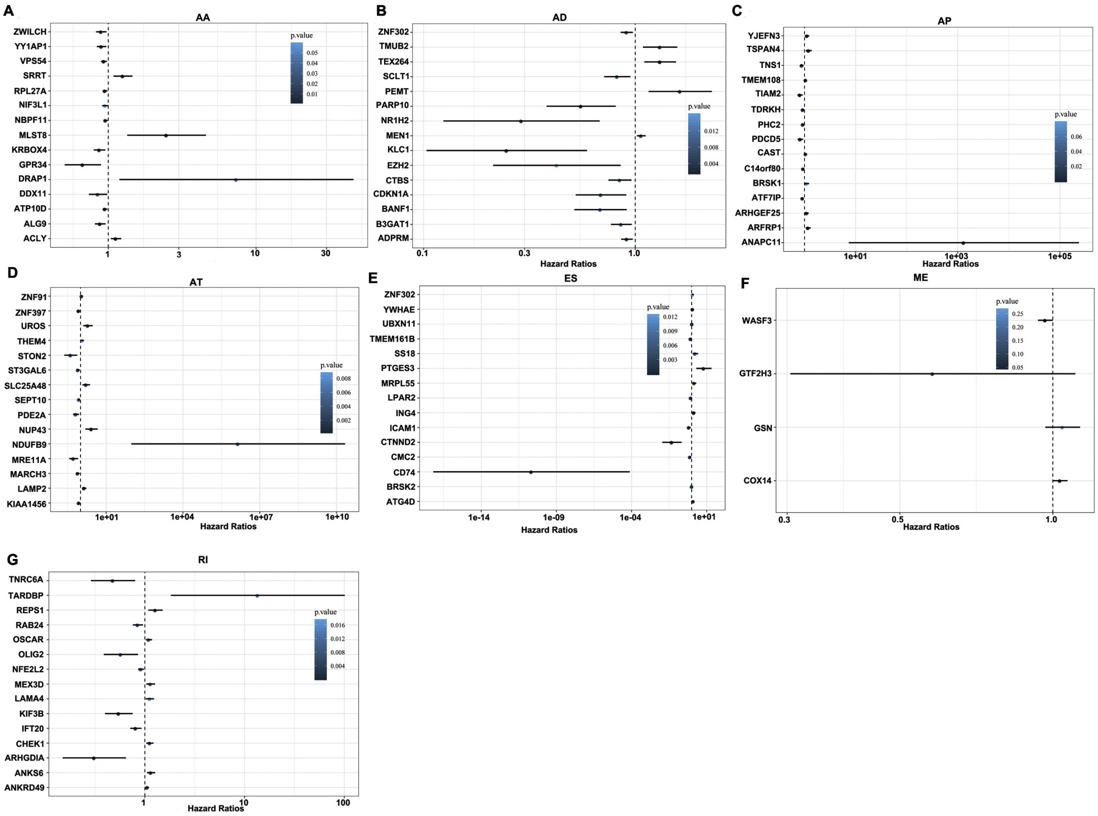

Supplementary Figure 4. Forrest plots of recurrence-associated AS events in GBM. A - G. Hazard ratios of top 10 recurrence associated AA, AD, AP, AT, ES, ME, and RI events.

Supplementary Figure 5

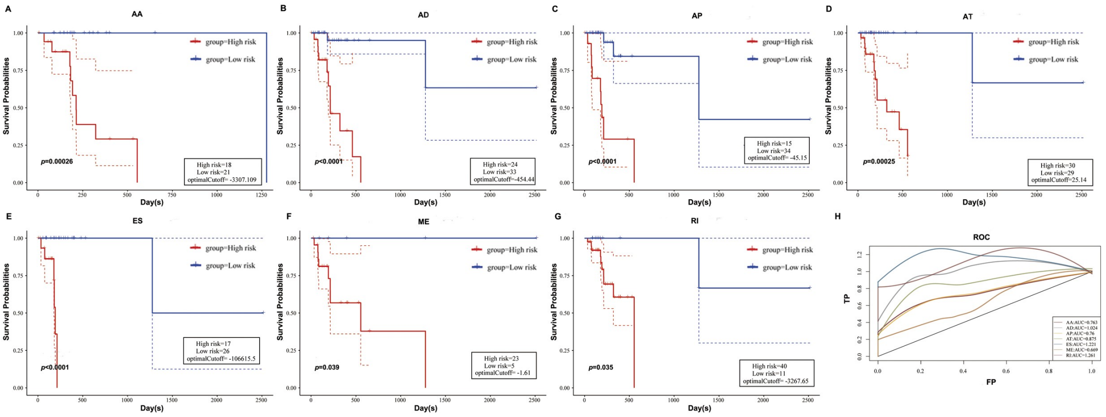

Supplementary Figure 5. Kaplan-Meier plots and ROC curves of prognostic predictor for GBM patients. A to G. Kaplan-Meier curves of prognostic predictor built with one type of recurrence associated AS events for GBM patients, respectively. Red line indicates high-risk group, while blue line indicates low-risk group. H. ROC curves with AUC of prognostic predictor built by one type of all seven types of recurrence-associated AS events in GBM.

Supplementary Figure 6

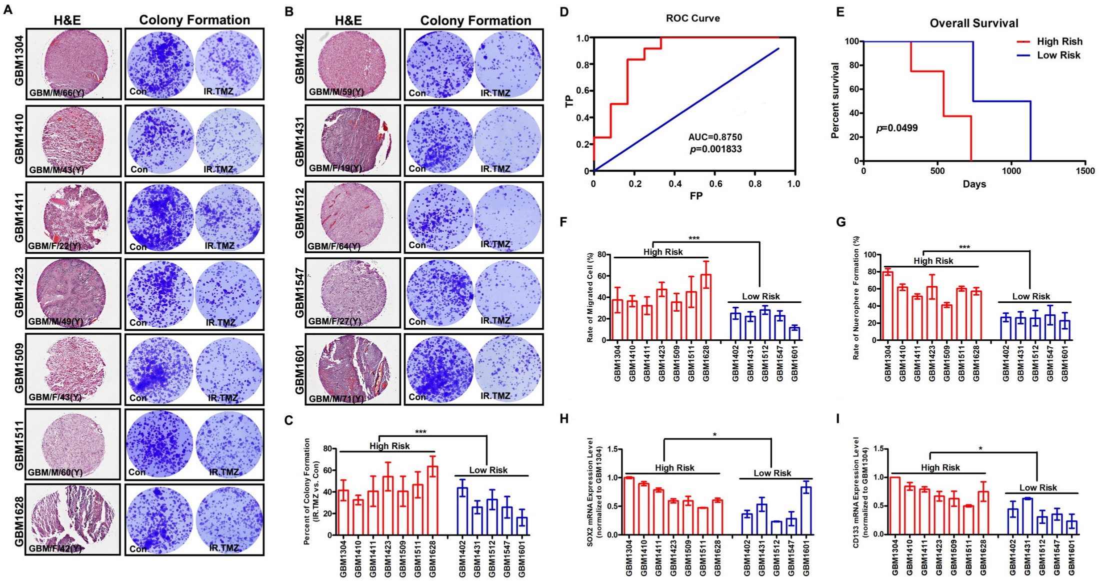

Supplementary Figure 6. Application in clinical practice of the final prognostic predictor on 5 feature genes. A-C. Colony formation assay for 12 GBM specimen-derived cells under radiotherapy (6 Gy) and temozolomide treatment (200 μM). D. ROC curve with AUC under the final prognostic predictor. E. The Kaplan-Meier curve of 12 GBM patients. Red line indicates high-risk group, while blue line indicates low-risk group, based on the final prognostic predictor. F. The proportion of migrated GBM cells derived from GBM patients. G. The rate of neurosphere formation for GBM cells detrived from GBM patients. H and I, the SOX2 (H), or CD133 (I) mRNA level of GBM cells detrived from GBM patients determined by RT-PCR. Data are presented as means ± SEM (*, p < 0.05; **, p < 0.01; ***, p < 0.001).
